# Analysis of immune cell infiltration in the tumor microenvironment of cervical cancer and its impact on immunotherapy

**DOI:** 10.3389/fonc.2025.1608597

**Published:** 2025-07-03

**Authors:** Fei Qin, Lu Huan, Xia Shi, Yuanyuan Hua, Yi Wu

**Affiliations:** ^1^ Department of Gynecology and Obstetrics, The Second Affiliated Hospital of Chongqing Medical University, Chongqing, China; ^2^ Renji Hospital, School of Medicine, Chongqing University, Chongqing, China

**Keywords:** cervical cancer, multi-omics analysis, immune infiltration, tumor mutation burden, tumor microenvironment

## Abstract

**Background:**

Cervical cancer remains a leading cause of cancer-related mortality among women worldwide. Despite advances in vaccination and early screening, late-stage diagnoses are common and associated with poor outcomes. This study aimed to identify novel prognostic biomarkers and therapeutic targets through a multi-omics approach, providing insights into the tumor immune microenvironment.

**Methods:**

We integrated transcriptomic, mutational, and clinical data from The Cancer Genome Atlas (TCGA) and Gene Expression Omnibus (GEO) to construct a prognostic model. Differential gene expression, enrichment analysis, immune infiltration profiling, and drug response prediction were performed to explore molecular features and therapeutic relevance.

**Results:**

Key high-risk biomarkers (EZH2, PCNA, BIRC5) and protective factors (CD34, ROBO4, CXCL12) were identified. The model effectively stratified patient survival in both cohorts and showed strong predictive performance. High-risk patients displayed distinct immune cell infiltration patterns and upregulated immune checkpoint expression, suggesting potential benefit from immunotherapy. Additionally, higher tumor mutational burden (TMB) was associated with improved survival. Drug sensitivity analysis indicated increased responsiveness of high-risk patients to agents such as Afuresertib and Venetoclax.

**Conclusion:**

This study establishes a reliable prognostic model and identifies critical biomarkers associated with cervical cancer progression, offering valuable insights into personalized therapeutic strategies. The findings contribute to a more comprehensive understanding of the disease and provide a foundation for future clinical applications. Nevertheless, further large-scale validation is required to confirm these findings and enhance their clinical utility.

## 1 Introduction

Cervical cancer presents a significant global public health issue for women, as it stands as the fourth most common gynecological malignancy, thereby representing a major health concern on an international level (1). In 2020, approximately 604,000 new instances of cervical cancer were reported, resulting in around 342,000 deaths worldwide attributed to this malignancy (2). The predominant contributors to cervical cancer are widely acknowledged to be persistent infection with human papillomavirus (HPV) and the failure to eliminate the virus effectively (3). The timely administration of the HPV vaccine has proven to be an efficient preventative measure against cervical cancer (4, 5). However, it is important to note that this vaccine does not eradicate existing HPV infections (6). Furthermore, current treatment modalities, which include a combination of established methods such as radiotherapy, chemotherapy, and surgical excision, are often hampered by undesirable side effects and show limited efficacy in managing advanced disease stages (7). Despite progress in vaccination and early detection techniques, the rate of cervical cancer remains alarmingly elevated, particularly among patients diagnosed at later stages, who exhibit poor survival rates. This underscores an urgent need for innovative diagnostic and therapeutic strategies to enhance patient outcomes.

Recent research underscores the complex interactions between cancer and the immune microenvironment, indicating that both immune cells and tumor cells participate in dynamic exchanges that significantly affect tumor progression (8–10). A disruption in these interactions plays a pivotal role in tumorigenesis, with all cancer types displaying a shared characteristic of evading immune detection (11). Within this changing context, immunotherapy has emerged as a notable breakthrough, especially following the sanctioning of programmed cell death protein 1 (PD-1) inhibitors for the treatment of recurrent or metastatic cancers (12). Specifically, the US Food and Drug Administration (FDA) approved pembrolizumab for patients with recurrent and advanced-stage cervical cancer in 2021 (13). Additionally, the European Medicines Agency (EMA) made a similar decision in 2022, endorsing Cemiplimab due to its significant effect on overall survival rates in cervical cancer patients, which revealed a median survival of 12.0 months versus 8.5 months, accompanied by a hazard ratio of 0.69 (14). This highlights the promise of immunotherapeutic approaches in addressing the immunological evasion tactics employed by tumors. However, existing analyses often focus on individual omics datasets, lacking a robust integration of multi-omics methodologies. Thus, there is an urgent need for a thorough examination of various omics dimensions—including genomics, transcriptomics, and proteomics—to enhance our understanding of the molecular characteristics and underlying mechanisms of cervical cancer. By leveraging multi-omics data, researchers aspire to identify novel prognostic biomarkers and therapeutic targets that could facilitate personalized treatment approaches and improve clinical outcomes for patients.

Despite growing interest in immune-related prognostic models for cervical cancer, existing studies still exhibit key limitations. For instance, Yao et al. (15) constructed a transcriptomics-based immune score but did not incorporate genomic mutations or drug sensitivity. Chen et al. (16) focused on immune subtypes and ICI scores but lacked interpretable modeling of individual gene contributions. Lin et al. (17) proposed a predictive signature for immunotherapy efficacy, yet omitted tumor mutational burden (TMB) and multi-omics integration. Similarly, Wang et al. (18) analyzed immune-related lncRNA pairs, but their work remained limited to transcriptomic data with no exploration of model transparency. In contrast, our study addresses these limitations by establishing a comprehensive and interpretable model that integrates multi-omics data and provides actionable insights into prognosis and therapy.

To address these gaps, we developed a comprehensive and interpretable prognostic model for cervical cancer by integrating transcriptomic, mutational, and clinical data from TCGA and GEO cohorts. Our approach incorporates SHapley Additive exPlanations (SHAP) to assess the contribution of each gene to risk prediction, thereby enhancing the transparency and biological interpretability of the model. Beyond model construction, we further examined the relationship between the SHAP-defined risk score and tumor immune cell infiltration, tumor mutational burden (TMB), and potential response to immune checkpoint inhibitors and chemotherapeutic agents. This integrative framework not only improves the accuracy of prognostic prediction but also provides valuable insights into individualized treatment strategies and potential therapeutic targets for cervical cancer patients.

## 2 Materials and methods

### 2.1 Data available source

The expression profiles along with clinicopathological parameters were sourced from The Cancer Genome Atlas (TCGA). Furthermore, the Gene Expression Omnibus (GEO) dataset GSE30759 was employed for our analysis (19, 20). Relevant URLs for online resources and analytical tools have been carefully included in the context to facilitate convenient access.

### 2.2 Data collection and differentially expressed genes

Gene expression, clinical, and mutation datasets were sourced from TCGA repository. Notably, the DEG analysis in this study utilized the paired tumor-normal tissue samples available in TCGA, but did not incorporate normal tissue data from the Genotype-Tissue Expression (GTEx) project, which provides a broader reference for gene expression in healthy tissues. The expression data were available in Fragments Per Kilobase of transcript per Million mapped reads (FPKM) format and were log_2_-transformed using the formula log_2_(FPKM + 1) prior to downstream analysis to stabilize variance and improve comparability. Clinical details, including patient demographics, tumor staging, treatment regimens, and survival data—were also extracted. Additionally, the GEO database, part of the National Center for Biotechnology Information (NCBI), was queried using the term “Cervical Cancer Survival” to obtain relevant datasets, notably GSE30759. GSE30759 was selected after stringent criteria, including a large sample size (n=292), consistent clinical staging (covering stages I-IV), and technical alignment with TCGA (Affymetrix GPL570 platform), to ensure cross-cohort comparability in gene expression profiling. Each dataset encompassed clinical sample information reflecting a range of disease states, and the GPL platform included gene probes associated with the expression matrix. Subsequently, the total sample count and staging for each dataset were compiled and cataloged. For the GSE30759 dataset, quantile normalization was performed using the normalizeBetweenArrays function in the limma package to ensure consistency in expression scale. Importantly, the TCGA and GEO datasets were used separately as the training and validation cohorts, respectively; thus, batch effect correction across datasets was not applied. This design avoids artificial variance introduced by inter-cohort integration and enables robust external validation. The limma R package was utilized to determine DEGs between tumor and normal tissue samples across both TCGA and GEO datasets. Statistical evaluations were performed using either t-tests or ANOVA, contingent on the distribution of the data. Genes were deemed significantly differentially expressed if they satisfied the criteria of |log_2_ fold change| > 1 and a p-value < 0.05.

### 2.3 Functional enrichment analysis

In order to investigate the biological functions associated with the DEGs identified, we conducted enrichment analyses utilizing Gene Ontology (GO) and the Kyoto Encyclopedia of Genes and Genomes (KEGG) pathways through the clusterProfiler R package. Significance was determined based on a p-value threshold of less than 0.05. The HALLMARK gene set utilized in our study was sourced from the MSigDB database, and the Gene Set Enrichment Analysis (GSEA) was executed on the differentially expressed genes distinguishing high-risk from low-risk groups, also employing the “clusterProfiler” package. For visualization purposes, the “GseaVis” R package was implemented.

### 2.4 Prognostic genes and model

A univariate Cox regression analysis was conducted utilizing the survival R package to pinpoint genes linked to prognosis. Genes exhibiting a p-value of less than 0.05 were designated as potential prognostic biomarkers. Subsequently, these candidates underwent further validation via multivariate Cox regression analysis to ascertain independent prognostic factors. To create a prognostic risk model, the Least Absolute Shrinkage and Selection Operator (LASSO) regression technique was employed, which aids in the selection of crucial prognostic genes while mitigating the risk of overfitting. The glmnet R package facilitated the construction of the model. The risk score for each patient was computed based on the following formula:

Risk Score=∑i=1n(Coefficient of Genei×Expression Level of Genei)\text{Risk Score = \sum_{i=1}^{n} (\text{Coefficient of Gene}_i \times \text{Expression Level of Gene}_i)Risk Score=i=1∑n​(Coefficient of Genei​×Expression Level of Genei​).

Moreover, to determine whether the risk score was an independent prognostic factor, multivariate Cox regression analysis was conducted while adjusting for clinical covariates such as age, gender, and tumor stage.

### 2.5 Model interpretation with SHapley Additive exPlanations analysis

In order to determine the importance of a specific attribution within the ensemble tree framework, the concept of gain is frequently utilized. Gain quantifies the overall reduction in loss that can be attributed to all the splits associated with the given attribution (21). However, the limitations of gain were emphasized by Lundberg, Erion, and Lee (22). To address these issues, Lundberg (23) introduced the Shapley value to measure the importance of various predictors. This approach suggests that the relevance of a specific predictor might diminish, even when the models increasingly rely on it. Named after the economist who proposed it, the Shapley value provides an equitable method for distributing gains among multiple contributors based on their respective contributions (24).

### 2.6 Survival analysis, ROC curve, and nomogram construction

In order to develop the nomogram, a multivariate Cox proportional hazards regression analysis was executed, which integrated clinical parameters such as age, sex, tumor stage, tumor grade, and a calculated risk score. The model was fitted utilizing the “cph” function available in the “rms” package. The nomogram was constructed based on the regression coefficients obtained from the Cox model, aimed at forecasting overall survival (OS) at 1, 3, and 5 years. Each clinical variable was allocated a score reflecting its influence on the predictive model, and the total of these scores yielded an overall score that estimates the probability of survival. Additionally, Kaplan-Meier survival analysis was performed through the survival R package to evaluate the survival outcomes between high-risk and low-risk cohorts. Receiver Operating Characteristic (ROC) curves were generated, and the Area Under the Curve (AUC) was computed to evaluate the predictive accuracy of the model.

### 2.7 Immune cell infiltration analysis

The ESTIMATE R package (25) (https://r-forge.r-project.org/projects/estimate/, accessed on May 19, 2024) was employed to derive the sample matrix score, immune score, and tumor purity. Furthermore, immune cell infiltration and the tumor microenvironment (TME) were assessed utilizing the CIBERSORT (26), TIMER (27), and ssGSEA (28) algorithms. The relationship between the risk score and immune checkpoint expression was analyzed through the Tumor and Immune System Interaction Database (TISIDB) (http://cis.hku.hk/TISIDB, accessed on May 22, 2024). Additionally, various methods for analyzing immune infiltration were applied to investigate the composition and distribution of immune cells between high- and low-risk groups within TCGA datasets.

### 2.8 Tumor mutational burden analysis

TMB was assessed utilizing mutation data from TCGA and is characterized as the aggregate count of somatic mutations per megabase of genomic sequence. An evaluation of the correlation between TMB and patient outcomes was conducted. For each patient within both the training and validation cohorts, TMB was established from somatic mutation data, subsequently categorizing patients into high-risk and low-risk groups according to the median TMB value.

### 2.9 Drug sensitivity prediction, immune escape and immunotherapy response analysis

Data derived from TCGA were scrutinized to evaluate the mutation frequencies of genes within high-risk and low-risk patient cohorts. Subsequently, the TIDE (29) algorithm (http://tide.dfci.harvard.edu/, accessed on May 26, 2024) was employed to predict the response of risk scores to Immune Checkpoint Inhibitors (ICIs). In addition, the Genomics of Drug Sensitivity in Cancer (GDSC) database (https://www.cancerrxgene.org/, accessed on May 28, 2024) was leveraged to assess the sensitivity of individual samples to various chemotherapy agents, utilizing the pRRophetic package to analyze and compare the half-maximal inhibitory concentration (IC50) values of these pharmacological agents (30, 31) (https://github.com/paulgeeleher/pRRophetic, accessed on May 28, 2024).

### 2.10 Statistical analysis

Survival disparities across different risk categories were evaluated utilizing Kaplan-Meier (KM) survival analysis. Predictive models were constructed employing both LASSO regression and Cox regression methodologies. All data processing and statistical evaluations were conducted using R software (version 4.3.2), with a significance threshold established at p < 0.05. Additionally, significance levels were defined as follows: p < 0.05 (*), p < 0.01 (**), and p < 0.001 (***). A p-value < 0.05 was chosen for analyzing drug sensitivity.

### 2.11 Single-cell RNA-seq analysis and intercellular communication inference

Single-cell RNA sequencing data from cervical cancer samples were obtained from the GEO database (GSE168652) (32). Raw count data were processed using the Seurat R package (v4.3.0). Cells with fewer than 50 detected genes or over 5% mitochondrial gene expression were excluded. The data were log-normalized, and 1,500 highly variable genes were identified. Dimensionality reduction was performed via PCA and t-SNE, followed by clustering using a shared nearest neighbor (SNN) algorithm with a resolution of 0.5. Marker genes were identified using the Wilcoxon rank-sum test (logFC > 1, adjusted p < 0.05). Cell types were annotated using the SingleR package with the Human Primary Cell Atlas as a reference, supplemented by manual validation based on canonical markers. The expression patterns of key prognostic genes (EZH2, CXCL12, PCNA, BIRC5, et al.) were assessed across clusters using violin plots, dot plots, and feature scatter maps. To investigate intercellular communication, CellPhoneDB (v2.1.7) was employed to predict significant ligand–receptor interactions among cell types. Interaction frequency and strength were visualized, highlighting key immunomodulatory pathways such as LGALS9–CD44. All analyses were performed using standard functions in Seurat, SingleR, and CellPhoneDB.

## 3 Results

### 3.1 Differential gene expression highlights immune-related alterations in the tumor microenvironment and enrichment analysis

**Figure 1:**
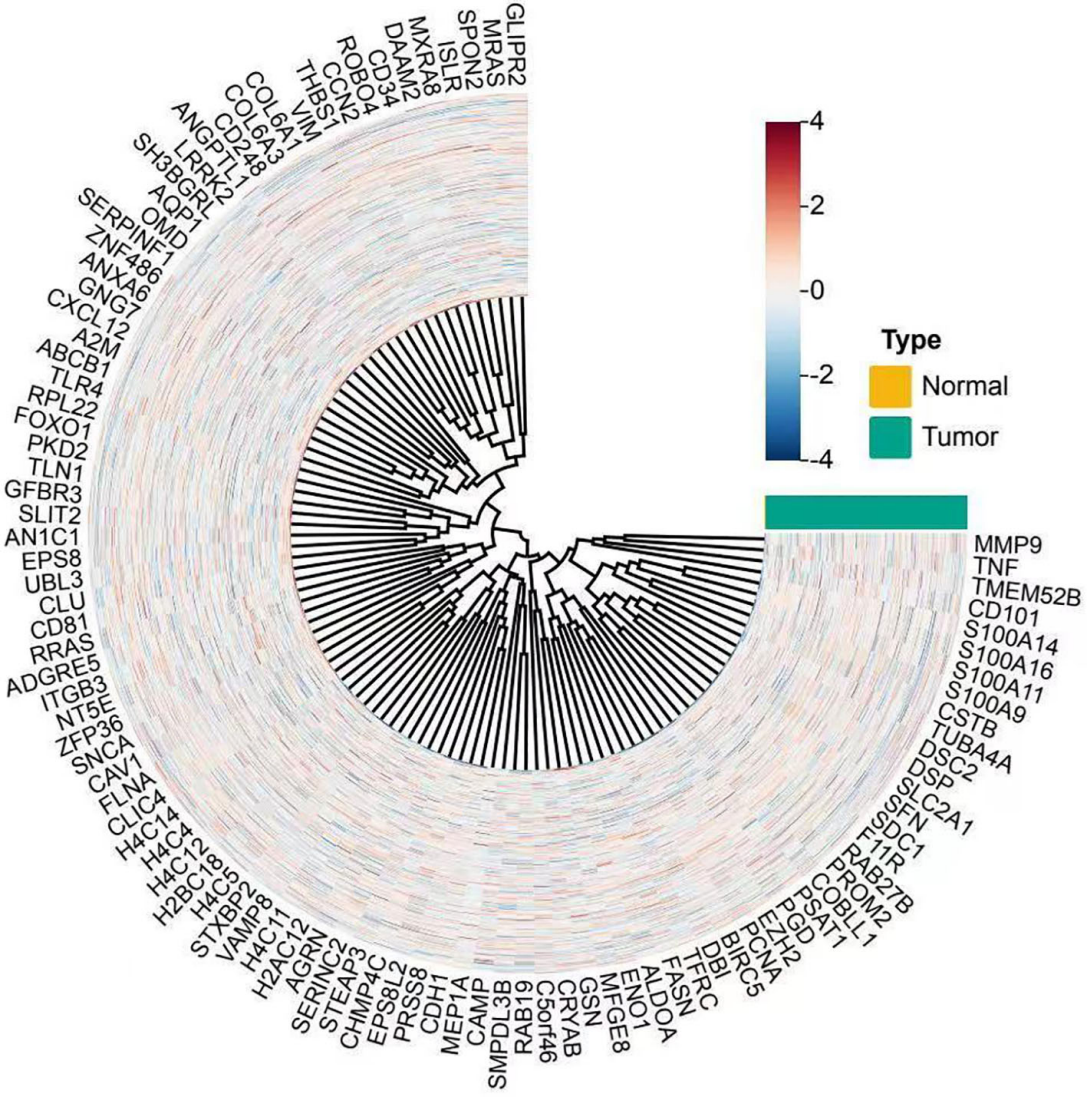
Circular heatmap showing differential gene expression between norml and tumor cervical tissue samples. Each spoke represents a gene, and each ring represents a sample. Samples are annotated by type, with normal tissues in yellow and tumor tissues in teal. Gene expression values are normalized and scaled (z-score), with red indicating higher and blue indicating lower expression levels. The clustering reveals distinct gene expression profiles between tumor and normal tissues, with upregulated genes such as MMP9, TNF, and S100A family prominently clustered in tumor samples.

To investigate the molecular characteristics associated with immune cell infiltration in cervical cancer, we conducted differential gene expression analysis between tumor and normal cervical tissue samples. As shown in **Figure 1**, the circular heatmap demonstrated a distinct separation between the two groups. Tumor samples exhibited significantly elevated expression of multiple immune- and inflammation-related genes, including MMP9, TNF, and members of the S100A family. These genes were previously reported to be involved in immune activation, cytokine signaling, and remodeling of the tumor microenvironment. The observed clustering pattern suggested that cervical cancer tissues possessed a distinct transcriptional profile enriched in immune-associated pathways, which may contribute to the altered immune cell composition and influence the effectiveness of immunotherapy.

**Figure 2:**
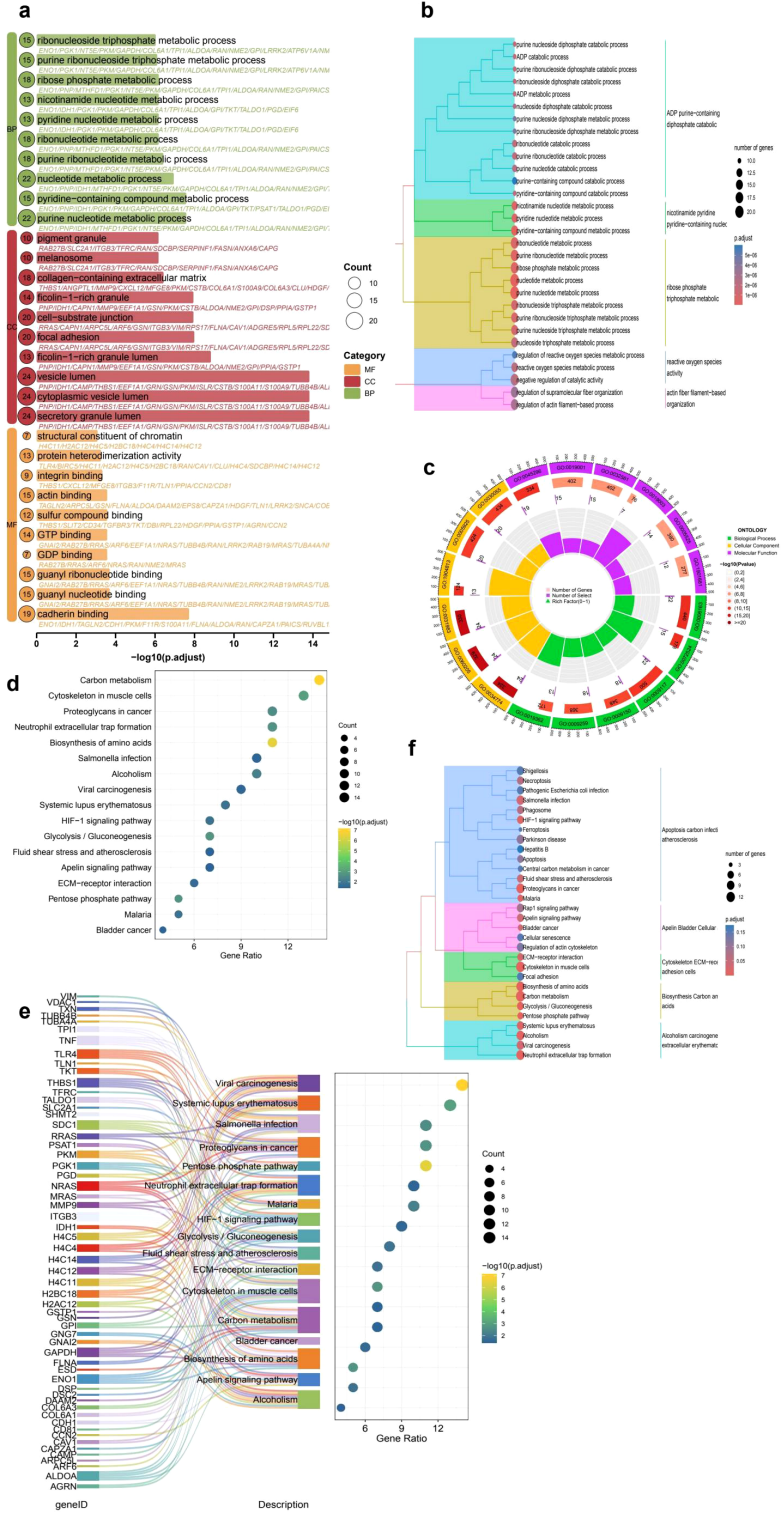
Functional enrichment analysis of differentially expressed genes (DEGs) in cervical cancer. **(a)** GO enrichment bubble plot displaying top significantly enriched terms across Biological Process (BP), Cellular Component (CC), and Molecular Function (MF) categories. Bubble size represents the number of genes, and color indicates the GO category. **(b)** Hierarchical clustering of enriched GO terms illustrating the relationship among biological processes. **(c)** Circular visualization of GO categories, highlighting the functional distribution and classification of enriched terms. **(d)** KEGG pathway enrichment analysis showing key tumor-related and immune-associated pathways such as ECM-receptor interaction, HIF-1 signaling, and proteoglycans in cancer. **(e)** Sankey plot linking DEGs to enriched KEGG pathways, demonstrating the multifunctional involvement of core genes in immune and metabolic processes. **(f)** Hierarchical clustering of KEGG pathways demonstrating modular associations among functionally related processes, including glycolysis/gluconeogenesis, neutrophil extracellular trap formation, and extracellular matrix remodeling, which may jointly influence tumor progression and immune dynamics.

To further explore the biological significance of DEGs in the context of immune cell infiltration, we performed GO and KEGG enrichment analyses. GO analysis (**Figures 2a–c**) revealed that the DEGs were significantly enriched in immune-related biological processes such as nucleotide metabolic processes, pyridine-containing compound metabolism, and extracellular matrix organization. Many of these terms were also functionally clustered, as shown in the hierarchical dendrogram, suggesting coordinated regulation within immune and metabolic pathways. KEGG pathway analysis (**Figure 2d**) indicated significant enrichment in pathways associated with tumor progression and immune modulation, including the HIF-1 signaling pathway, ECM-receptor interaction, and proteoglycans in cancer. The Sankey diagram (**Figure 2e**) further illustrated that several core genes were involved in multiple immune- and microenvironment-associated pathways, highlighting their potential role in shaping the tumor immune landscape and influencing immunotherapy responsiveness. To further explore the biological significance of DEGs in the context of immune cell infiltration, we performed GO and KEGG enrichment analyses. GO analysis (**Figures 2a–c**) revealed that the DEGs were significantly enriched in immune-related biological processes such as nucleotide metabolic processes, pyridine-containing compound metabolism, and extracellular matrix organization. Many of these terms were also functionally clustered, as shown in the hierarchical dendrogram, suggesting coordinated regulation within immune and metabolic pathways. KEGG pathway analysis (**Figure 2d**) indicated significant enrichment in pathways associated with tumor progression and immune modulation, including the HIF-1 signaling pathway, ECM-receptor interaction, and proteoglycans in cancer. The Sankey diagram (**Figure 2e**) further illustrated that several core genes were involved in multiple immune- and microenvironment-associated pathways, highlighting their potential role in shaping the tumor immune landscape and influencing immunotherapy responsiveness.

### 3.2 Clustering analysis of risk groups and prognosis-related differential genes

**Figure 3:**
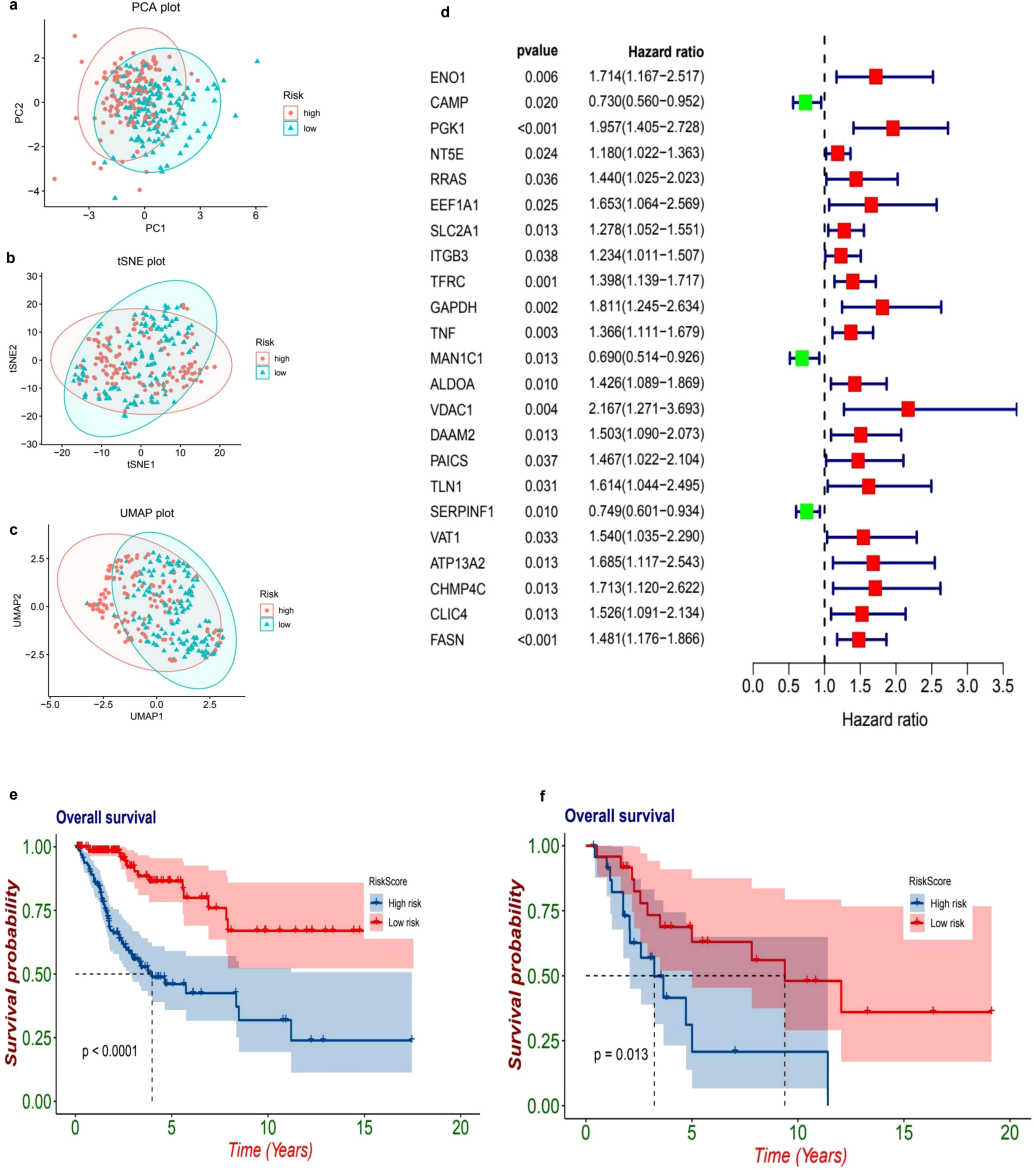
Risk stratification and survival analysis of patients based on gene expression profiles. The dimensionality reduction plots illustrate the distribution of patients based on their risk levels. In the first plot **(a)**, a Principal Component Analysis (PCA) is presented, showcasing how patients categorized as high-risk (marked in red) and low-risk (marked in blue) are distributed across the first two principal components, PC1 and PC2. Following this, the t-Distributed Stochastic Neighbor Embedding (t-SNE) plot **(b)** visualizes the separation of these high-risk and low-risk groups based on their gene expression profiles, highlighting distinct clusters. The Uniform Manifold Approximation and Projection (UMAP) plot **(c)** further emphasizes the clustering of high-risk and low-risk groups in a two-dimensional space, providing additional clarity on their separation. Moving to the forest plot **(d)**, this univariate Cox regression analysis presents hazard ratios (HR) and 95% confidence intervals (CI) for key genes linked to patient prognosis. In this plot, red squares indicate significant high-risk genes (with HR greater than 1), while green squares represent protective genes (with HR less than 1). The Kaplan-Meier survival curves **(e, f)** provide further insights into patient outcomes, with plot **(e)** comparing overall survival rates between high-risk (red) and low-risk (blue) patients, revealing a significant difference (p < 0.001) where high-risk patients show poorer survival outcomes. Lastly, plot **(f)** presents a subgroup survival analysis that reinforces the significant survival difference (p = 0.024) between the risk groups, further validating the prognostic value of the risk model.

The Principal Component Analysis (PCA) visualization reveals a prominent separation between high-risk and low-risk cohorts across the principal components, indicating inherent variability in gene expression profiles (**Figure 3a**). In a similar vein, both t-SNE and UMAP (**Figures 3b, c**) visualizations exhibit effective clustering, thereby reinforcing the notion that the model is capable of distinguishing between risk categories based on gene expression patterns. The prognostic evaluation uncovered numerous differentially expressed genes linked to survival outcomes. Notable genes such as ENO1, PGK1, NT5E, and RRAS displayed hazard ratios exceeding 1, implying potential risk correlations. In contrast, genes CAMP and MAN1C1 presented hazard ratios lower than 1, suggesting possible protective roles (**Figure 3d**).

### 3.3 Prognostic model

The datasets from TCGA and GEO have been classified into high-risk and low-risk categories utilizing a predetermined cutoff threshold. Simultaneously, TCGA serves as the training dataset, while GEO functions as the validation dataset. An examination of the TCGA dataset indicates a marked difference in overall survival rates between the high-risk and low-risk cohorts, with a p-value that is less than 0.001 (HR = 0.2, 95% CI: 0.11 - 0.36, p = 6.53e-08. **Figure 3e**). Similarly, the GEO dataset demonstrates a significant difference in survival outcomes between these two categories, yielding a p-value of 0.013 (HR = 0.34, 95% CI: 0.13 - 0.91, p = 0.0313. **Figure 3f**), thereby reinforcing the validity of the prognostic model.

### 3.4 Independent prognostic analysis and model performance evaluation

**Figure 4:**
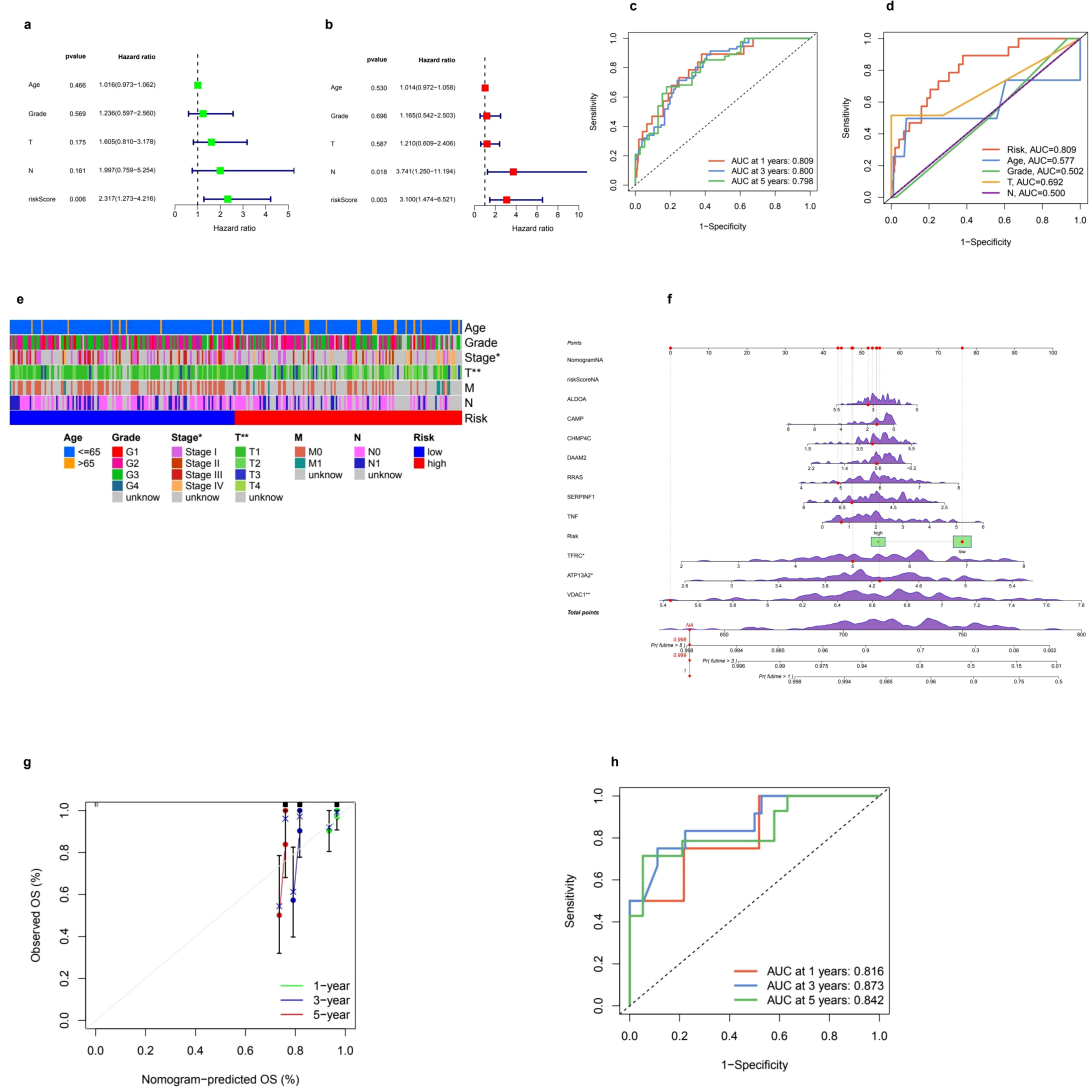
Prognostic model performance and clinical correlations. **(a, b)** Univariate and multivariate Cox regression analyses were conducted to evaluate clinical variables such as age, grade, TNM staging, and risk score in relation to overall survival (OS). The univariate analysis provided hazard ratios (HR) along with 95% confidence intervals (CI) for these variables. In the multivariate analysis, independent prognostic factors for OS were identified, confirming that the risk score remains a significant predictor even after adjusting for other clinical factors. **(c)** Additionally, receiver operating characteristic (ROC) curves were utilized to assess the prognostic performance of the model over time. The time-dependent ROC curves illustrated the model's predictive ability at 1, 3, and 5 years, accompanied by corresponding area under the curve (AUC) values. **(d)** Furthermore, ROC curves were compared to evaluate the predictive accuracy of the risk score against clinical parameters, highlighting the risk score's superior prognostic value. **(e)** A clinical heatmap was generated to visualize the distribution of clinical characteristics, including age, grade, and TNM stage, across low-risk and high-risk groups, with different colors representing various categorical clinical features. The mutation landscape of key prognostic genes was depicted through a waterfall plot, where red markers indicated significant mutations and green boxes highlighted key mutation hotspots. **(f)** Lastly, the calibration and validation of the nomogram were assessed, with a calibration plot illustrating the accuracy of the nomogram's predicted OS at 1, 3, and 5 years; **(g)** points closer to the diagonal line indicated better predictive performance. **(h)** The ROC curve for nomogram validation further demonstrated its discriminative power at the specified time points of 1, 3, and 5 years.

The univariate analysis highlights a notable correlation between the riskScore and patient prognosis (p = 0.006), with a calculated hazard ratio of 2.317 (**Figure 4a**). Additionally, the multivariate analysis corroborates the significance of both riskScore (p = 0.003) and T stage (p = 0.018) as independent prognostic indicators, each exhibiting hazard ratios of 3.100 and 3.741, respectively (**Figure 4b**). The time-dependent ROC analysis indicates that the predictive model consistently achieves an AUC of 0.809 (95% CI: 0.728–0.890) at one year, 0.800 (95% CI: 0.705–0.882) at three years, and 0.798 (95% CI: 0.694–0.875) at five years, reflecting its stable and dependable predictive capacity (**Figure 4c**). Furthermore, a comparative ROC analysis demonstrates that the risk model (AUC = 0.809) outperforms other clinical parameters, including Age (AUC = 0.577), Grade (AUC = 0.502), T stage (AUC = 0.692), and N stage (AUC = 0.500) (**Figure 4d**).

### 3.5 Clinical associations, nomogram construction, and predictive accuracy

The amalgamation of clinical parameters and prognostic indicators plays a pivotal role in evaluating patient outcomes. The clinical correlation heatmap illustrates the allocation of risk categories across a range of factors, including Age (≤65y, >65y), Grade (G1-G4, unknown), Stage (I-IV, unknown), T classification (T1-T4, unknown), M classification (M0, M1, unknown), and N classification (N0, N1, unknown). This stratification unveils notable associations between high-risk and low-risk groups with these clinical variables, highlighting their potential prognostic significance (**Figure 4e**). In addition, the nomogram functions as an advanced instrument that consolidates various prognostic factors to predict survival probabilities at intervals of 1, 3, and 5 years. Points are allocated based on individual risk profiles, thereby enhancing the precision of personalized predictions (**Figure 4f**). The robustness of this model is reinforced by calibration curves demonstrating a strong alignment between the predicted and actual survival rates, thereby affirming its credibility (**Figure 4g**). Furthermore, ROC curves reveal a high level of predictive accuracy, with AUC values recorded at 0.816, 0.873, and 0.842 for the 1-, 3-, and 5-year survival rates, respectively, showcasing the model’s superior discrimination ability (**Figure 4h**).

### 3.6 SHAP analysis

**Figure 5:**
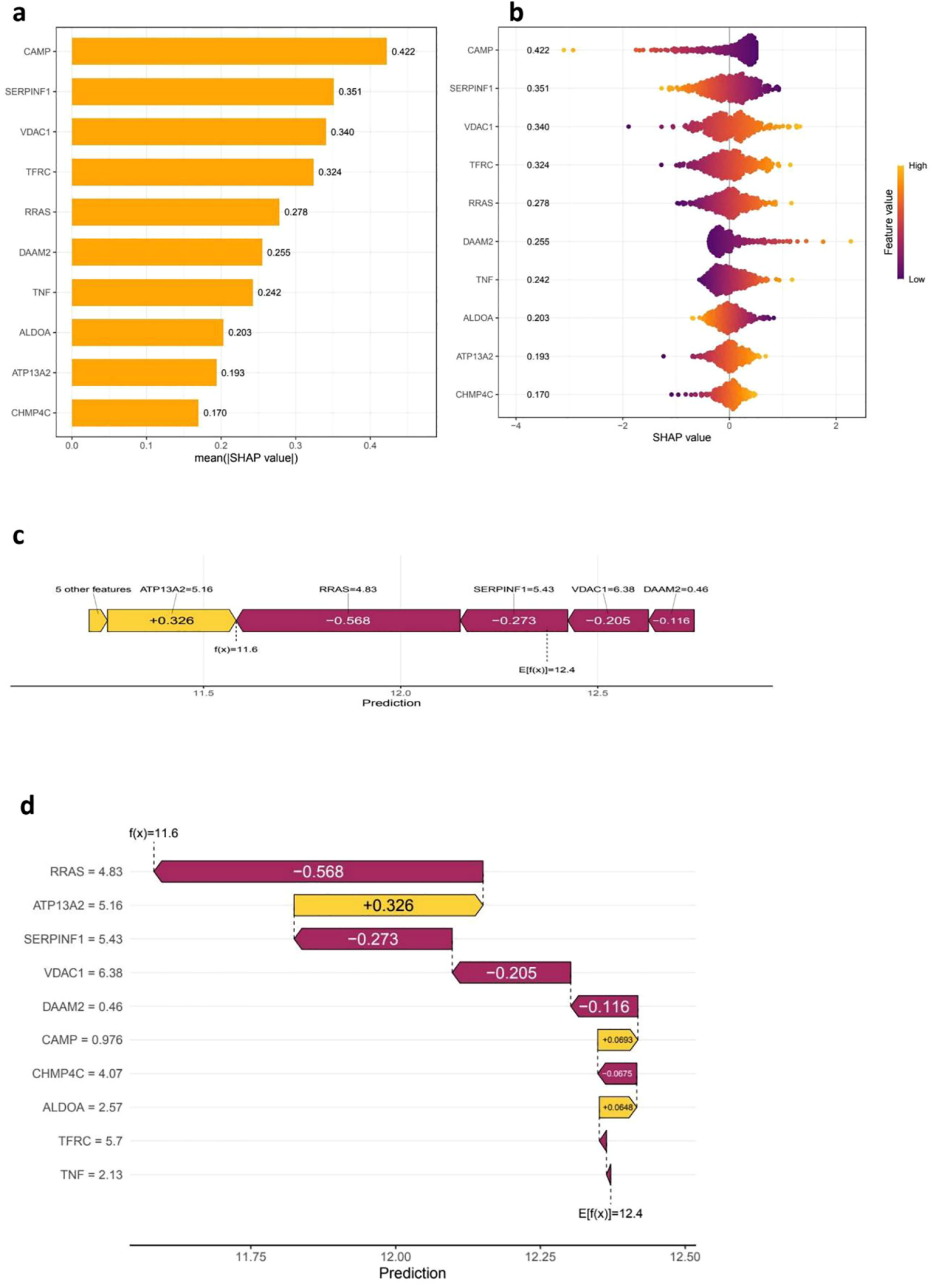
SHAP analysis for feature Importance and model interpretability. The **(a, b)** SHAP Summary Plots provide insights into feature importance by illustrating the mean absolute SHAP values, which reflect the overall contribution of each feature, or gene, to the model’s predictions. Features with higher mean absolute SHAP values are deemed more influential, with CAMP, SERPINF1, and VDAC1 identified as the top contributors. Additionally, the SHAP value distribution plot reveals how each feature impacts the model’s output, utilizing a color gradient to represent feature values—yellow indicates high values while purple signifies low values. This visualization highlights a clear trend in how the expression of these features influences risk predictions. Moving on to **(c, d)**, the SHAP Force and Waterfall Plots provide a closer look at individual predictions. The SHAP force plot illustrates how various features contribute to a specific patient’s prediction, where positive SHAP values increase the prediction, while negative values decrease the predicted risk score. The SHAP waterfall plot further breaks down the cumulative contribution of individual features to the final prediction score, detailing the model’s decision-making process and showcasing the relative impact of genes such as RRAS, ATP13A2, and SERPINF1 on the final outcome.

The SHAP analysis offers critical insights into the significance of features and their contributions to the predictive capabilities of the model. The accompanying bar plot (**Figure 5a**) clearly illustrates that features such as CHMP4C, ATP13A2, and ALDOA exhibit the highest mean absolute SHAP values, thereby signifying their substantial impact on the model’s predictions. Further examination through the bee plot (**Figure 5b**) delineates the relationship between varying feature values—especially for CHMP4C and ATP13A2—and their corresponding SHAP values, underscoring their pivotal roles in influencing the model’s outcomes. The force plot (**Figure 5c**) provides a detailed breakdown of an individual prediction, revealing that VDAC1, with a SHAP value of 6.38, alongside DAAM2, which contributes a lesser amount of 0.46, exerts significant positive influences that drive the prediction closer to its final value of 11.6. In a similar vein, the waterfall plot (**Figure 5d**) conveys the cumulative impact of each feature, showcasing how elements such as TNF, TFRC, and ALDOA either positively or negatively affect the predicted outcome, thereby highlighting their critical importance in the decision-making process of the model.

### 3.7 Immune cell infiltration and functional alterations

**Figure 6:**
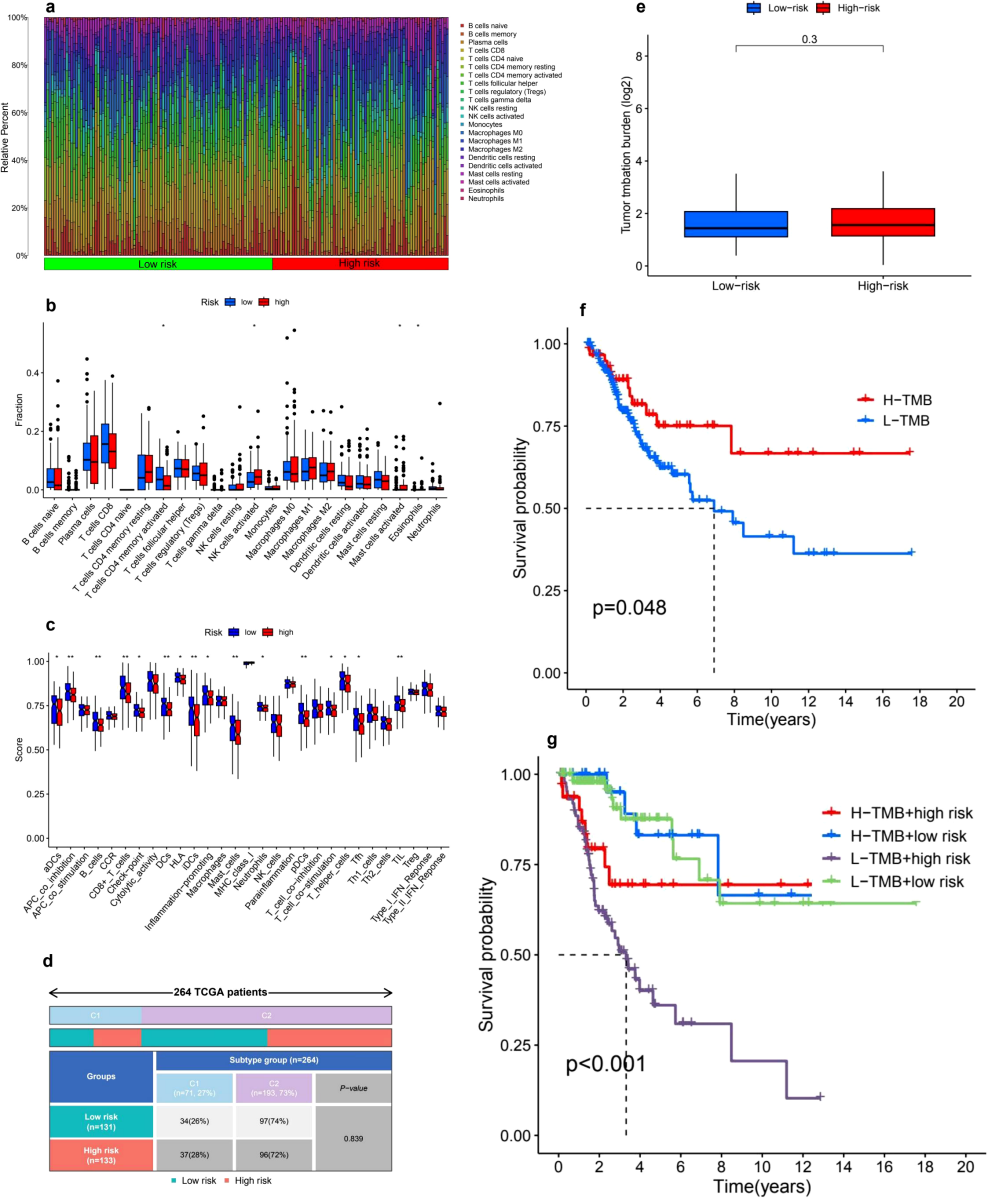
Tumor microenvironment and tumor mutation burden (TMB) analysis. **(a)** The analysis of immune cell infiltration distribution reveals notable differences between high-risk and low-risk groups, highlighting the relative abundance of various immune cell types. The color gradient effectively illustrates the diversity of immune cell populations across the samples. **(b)** A boxplot comparison of immune cell fractions between these two risk groups indicates significant differences in immune infiltration levels, with asterisks marking statistically significant differences (p < 0.05, p < 0.01, p < 0.001). **(c)** Furthermore, immune-related functional scores comparing different immune pathways between high-risk and low-risk patients uncover distinct patterns of immunological activity. **(d)** The distribution of risk groups among TCGA patient subtypes illustrates the proportions of low-risk and high-risk patients across various molecular subtypes. **(e)** In terms of tumor mutation burden (TMB), the comparison between the two risk groups shows no statistically significant difference (p = 0.3). **(f)** However, the Kaplan-Meier survival curve for high-TMB (H-TMB) and low-TMB (L-TMB) groups indicates that patients with higher TMB tend to experience better survival outcomes (p = 0.048). **(g)** Additionally, a combined survival analysis of TMB and risk groups reveals that the L-TMB + high-risk subgroup has the worst survival prognosis, while the H-TMB + low-risk subgroup demonstrates the best outcomes (p < 0.001).

Our investigation into the infiltration of immune cells and the functional variations between low- and high-risk cohorts revealed substantial differences in immune cell composition and associated processes (**Figure 6a**). In particular, we noted a significant enrichment of CD8+ T cells, M1 macrophages, and activated dendritic cells within one of the groups, indicating their pivotal involvement in tumor immunity. In contrast, M2 macrophages and naive B cells presented an opposing trend, which could suggest the existence of immunosuppressive mechanisms (**Figure 6b**). Additionally, we defined crucial immune functions that displayed marked differences between the two groups. These included the co-stimulation and inhibition of antigen-presenting cells (APCs), cytolytic activity, pro-inflammatory responses, and checkpoint activation (**Figure 6c**). The distinct patterns observed in T cell co-stimulation and inhibition, alongside type I/II interferon responses and the activity of macrophages and neutrophils, suggest potential imbalances in immunoregulation that might correlate with our risk classification. These results underscore the complex interactions of immune responses and their relevance in comprehending tumor microenvironments.

### 3.8 Immune subtype analysis

An immune subtype assessment was performed involving 264 patients from the TCGA cohort, categorized into two distinct immune subtypes: C1 and C2 (**Figure 6d**). The breakdown of immune subtypes within the low-risk and high-risk stratifications is as follows: Subtype C1 encompassed 71 patients (27%), comprising 34 patients (26%) in the low-risk category and 37 patients (28%) in the high-risk category. Conversely, Subtype C2 included 193 patients (73%), with 97 patients (74%) belonging to the low-risk group and 96 patients (72%) classified as high-risk. The statistical evaluation indicated that there were no significant differences in the distribution of immune subtypes between the low-risk and high-risk groups (P-value = 0.839).

### 3.9 TMB analysis

The comparison of TMB between low-risk and high-risk groups revealed no significant differences. The TMB values, displayed on a log2-transformed scale, were similar across both groups, suggesting that TMB may not serve as a distinguishing factor for different risk classifications (**Figure 6e**). However, Kaplan-Meier survival analysis indicated that patients with high TMB (H-TMB) experienced significantly better overall survival compared to those with low TMB (L-TMB), with a P-value of 0.048 (**Figure 6f**). Furthermore, when TMB status was integrated with risk classification, survival analysis showed significant differences among the four subgroups: H-TMB with high risk, H-TMB with low risk, L-TMB with high risk, and L-TMB with low risk, yielding a P-value of less than 0.001 (**Figure 6g**).

### 3.10 Immune evasion and drug sensitivity

**Figure 7:**
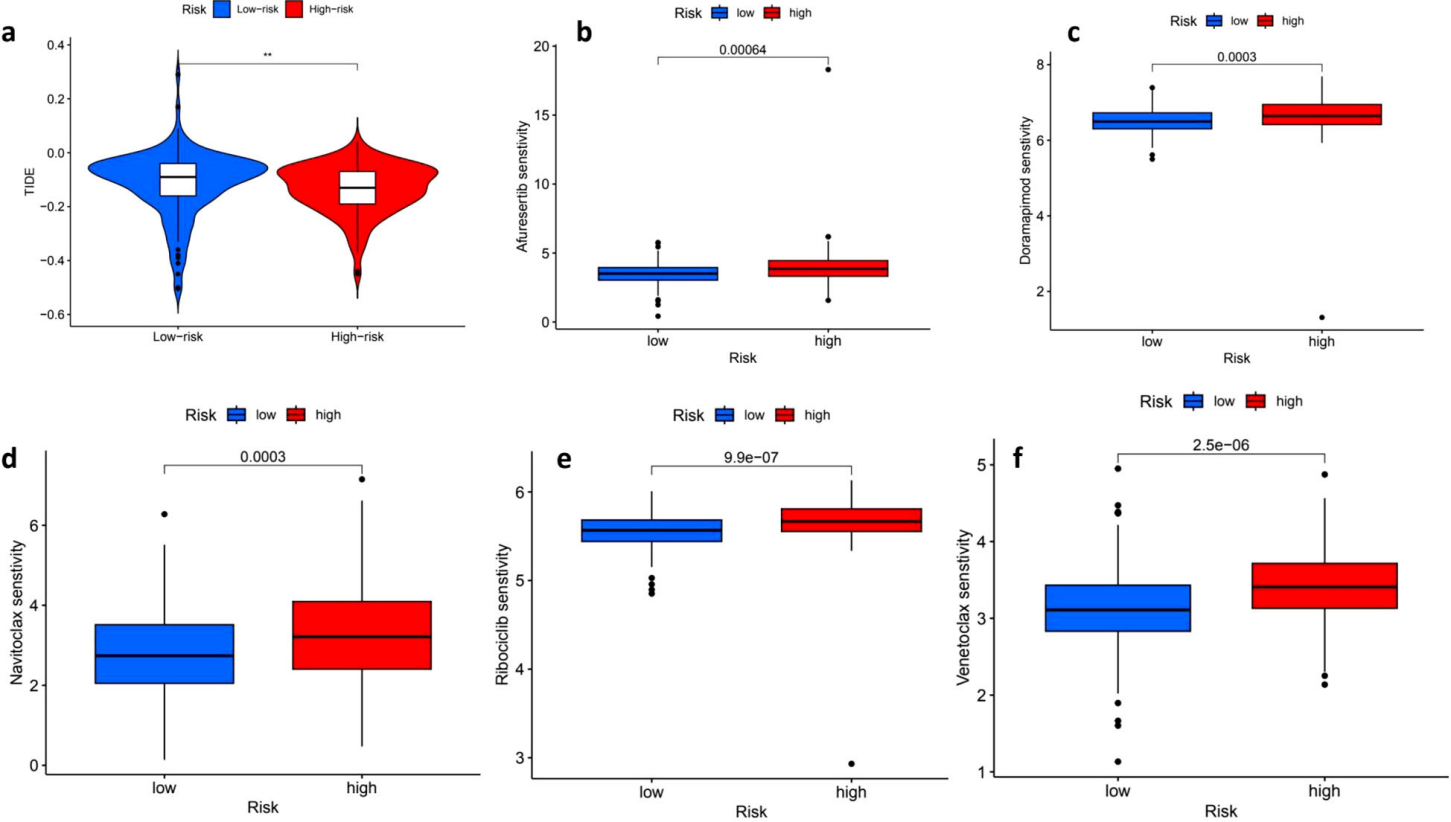
Tumor immune dysfunction and exclusion (TIDE) scores and drug sensitivity analysis. The distribution of Tumor Immune Dysfunction and Exclusion (TIDE) scores reveals a stark contrast between low-risk (blue) and high-risk (red) patients, with high-risk individuals exhibiting significantly elevated TIDE scores, which suggests a potential resistance to immune checkpoint blockade therapy (p < 0.01). In the context of drug sensitivity analysis across both high- and low-risk groups, several noteworthy findings emerged. For instance, the sensitivity to Alisertib was significantly greater in the high-risk group (p = 0.00064), while Doramapimod also demonstrated increased sensitivity among high-risk patients (p = 0.0003). Furthermore, a comparison of Navitoclax sensitivity indicated that high-risk patients had significantly higher drug sensitivity (p = 0.0003). Conversely, low-risk patients exhibited a higher sensitivity to Ribociclib, with a striking p-value of 9.9e-07. Lastly, the sensitivity to Venetoclax was significantly greater in high-risk patients as well, with a p-value of 2.5e-06, underscoring the complex dynamics of drug sensitivity in relation to patient risk stratification. ** means p<0.01.

The Tumor Immune Dysfunction and Exclusion (TIDE) analysis indicated that individuals in the high-risk group had elevated TIDE scores, which points to a greater potential for immune evasion and suggests they may have a poorer response to ICIs. Conversely, the low-risk group, characterized by lower TIDE scores, appears to be more likely to benefit from ICIs, making them potentially better candidates for immunotherapy (**Figure 7a**). Furthermore, the analysis of drug sensitivity revealed notable differences in how the low-risk and high-risk groups responded to various medications. Specifically, the high-risk group demonstrated increased sensitivity to several drugs, including Afuresertib (p = 0.00064) (**Figure 7b**), Doramapimod (p = 0.0003) (**Figure 7c**), Navitoclax (p = 0.0003) (**Figure 7d**), Ribociclib (p = 9.9e−07) (**Figure 7e**) and Venetoclax (p = 2.5e−06) (**Figure 7f**), indicating that the response to these treatments varies significantly based on the risk group.

### 3.11 Single-cell validation of prognostic genes and cell–cell communication patterns

**Figure 8:**
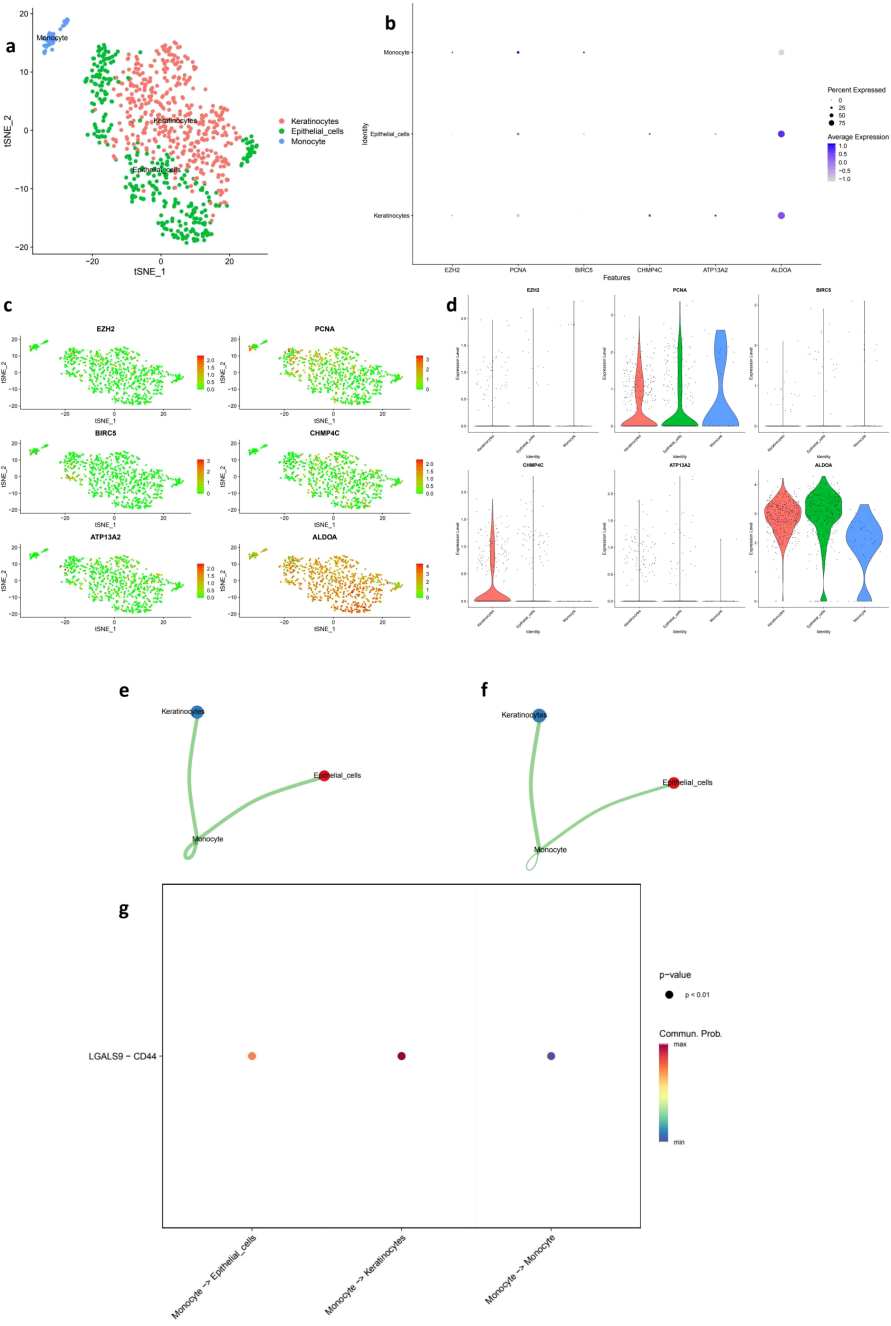
Single-cell transcriptomic validation and cell–cell communication analysis of prognostic genes. **(a)** t-SNE plot showing the clustering of single cells from the GSE168652 dataset into three major populations: keratinocytes, epithelial cells, and monocytes. **(b)** Dot plot of hub gene expression (EZH2, PCNA, BIRC5, CHMP4C, ATP13A2, ALDOA) across annotated cell types. Dot size represents the percentage of cells expressing each gene, and color indicates average expression level. **(c)** Feature plots illustrating the spatial distribution of hub gene expression across the t-SNE embedding. **(d)** Violin plots comparing the expression levels of hub genes among different cell populations. **(e)** Cell–cell communication frequency map showing the number of interactions among cell types, with stronger connectivity observed between monocytes and epithelial or keratinocyte populations. **(f)** Communication weight map reflecting the interaction intensity between cell types based on predicted ligand–receptor interactions. **(g)** Bubble plot displaying the statistically significant LGALS9–CD44 ligand–receptor interaction between monocytes and other cell types (p < 0.01), highlighting potential immunoregulatory mechanisms in the tumor microenvironment.

To validate our bulk RNA-seq findings, we reanalyzed the GSE168652 scRNA-seq dataset and identified three major cell populations: epithelial cells, keratinocytes, and monocytes (**Figure 8a**). Prognostic genes including EZH2, PCNA, and BIRC5 were specifically expressed in epithelial clusters, while CHMP4C, ATP13A2, and ALDOA showed broader expression patterns (**Figures 8b–d**), supporting their tumor-specific roles. CellPhoneDB-based communication analysis revealed strong interactions between monocytes and epithelial or keratinocyte cells (**Figures 8e, f**), with LGALS9–CD44 emerging as a key ligand–receptor pair (**Figure 8g**), suggesting potential immunoregulatory signaling. These single-cell results confirm the robustness of our signature genes, clarify their cellular origin, and reveal potential crosstalk within the tumor microenvironment that may influence immune response and therapeutic outcomes.

## 4 Discussion

Cervical cancer remains one of the most prevalent cancers among women worldwide, with annual statistics revealing more than 500,000 new cases and nearly 300,000 deaths attributed to the disease (33, 34). Our study employed comprehensive bioinformatics methods to analyze cervical cancer. Gene expression, clinical, and mutation data from TCGA and GEO were used to identify DEGs through the limma R package. Functional enrichment analysis (GO and KEGG) revealed key biological processes and pathways involved in cancer progression. A prognostic risk model was built using LASSO regression and validated with independent datasets. Notably, the validation cohort (GSE30759, n=292) was rigorously selected for its large sample size, consistent clinical staging (I-IV), and technical compatibility with TCGA (Affymetrix GPL570 platform), ensuring cross-cohort comparability. SHAP analysis transcended conventional model interpretation by quantifying the contribution of individual genes (CHMP4C, ATP13A2, ALDOA) to risk stratification, linking their expression to mechanistic pathways such as endosomal sorting (CHMP4C) and lysosomal function (ATP13A2), which may modulate immune evasion through impaired antigen presentation and exosome-mediated signaling. The model’s predictive power was confirmed through survival analysis, ROC curves, and nomogram construction. Additionally, immune cell infiltration analysis highlighted significant differences in the tumor microenvironment between high- and low-risk groups, while TMB and drug sensitivity predictions offered potential therapeutic insights. Together, these findings provide a comprehensive understanding of the molecular, immune, and clinical factors influencing cervical cancer prognosis and treatment.

The findings of this study provide important insights into the DEGs in cervical cancer and their potential impact on patient prognosis. Our results are consistent with previous research, confirming the presence of several well-established DEGs while also identifying new candidates that have not been widely reported in the literature. For example, genes like APOD, ACKR1, and SFRP4 have been recognized as significant in cervical cancer, and our analysis supports their involvement in tumor progression and patient survival, thereby reinforcing their potential as biomarkers in clinical practice. Furthermore, the prognostic model we developed demonstrated greater predictive power compared to those reported by Dong et al. (35, 36), primarily due to our larger sample size and the comprehensive integration of clinical and genomic data, highlighting the robustness of our findings. Multi-omics integration further revealed a molecular landscape characterized by dysregulated cancer metabolism (glycolysis/gluconeogenesis, pentose phosphate pathway) and immune response (HIF-1 signaling, systemic lupus erythematosus pathways), highlighting the interplay between metabolic reprogramming and immune evasion in tumor progression.

Our research identified several high-risk biomarkers, such as EZH2, PCNA, and BIRC5, alongside protective biomarkers like CD34, ROBO4, and CXCL12. Recognizing these biomarkers is crucial as they could assist in the early detection and tailored treatment strategies for patients with cervical cancer. Notably, EZH2 has been linked to tumor progression, and further exploration of its specific mechanisms may shed light on its role in cancer development and enhance our understanding of potential therapeutic targets (37). Moreover, the protective biomarkers could play a significant role in modulating the immune response, suggesting their relevance for immunotherapeutic approaches (33). The interplay of these biomarkers may enhance prognostic accuracy, highlighting the need for further research into their combined effects on predicting patient outcomes. Our established prognostic model demonstrated strong survival stratification capabilities across both the TCGA and GEO datasets, achieving impressive AUC values that underscore its reliability, with AUC values of 0.809 for 1-year, 0.800 for 3-year, and 0.798 for 5-year survival. This high level of accuracy underscores the model’s potential application in clinical settings, enabling personalized treatment strategies based on individual risk assessments. Future discussions should focus on the practical application of this model in clinical decision-making, particularly regarding its integration with other clinical indicators to improve predictive performance (38).

As previously acknowledged, CCL5 and CXCL10 serve as crucial signaling bridges between NK cells, T cells, and tumor cells (39). CXCL9, produced by myeloid cells activated through the STING pathway, acts as an intermediary that stimulates the secretion of IFN in T cells, which in turn enhances the expression of CXCL9 in myeloid cells. Furthermore, Li (40) emphasized the significant role of the STING pathway and the potential of MSA-2 in reshaping the immune microenvironment in cervical cancer. However, this study primarily focused on STING agonists and offered limited discussion on other immunotherapies. It also lacked comprehensive multi-omics analysis of immune infiltration and did not delve deeply into the mechanisms of adaptive immune escape. Therefore, gaining a thorough understanding of the interactions between immune microenvironments and therapeutic responses could aid in developing more effective clinical strategies tailored to individual patient profiles, particularly in relation to checkpoint inhibitor therapies (41). Our extensive study revealed a significant link between TMB and improved survival rates, highlighting TMB as a vital prognostic factor in cancer research. The correlation we found between TMB and immunogenicity indicates that patients with higher mutational loads are more likely to respond positively to immunotherapy. This underscores the importance of including TMB assessments in standard clinical practice to enhance patient outcomes. However, it is important to note that the overall TMB distribution did not show statistically significant differences between high-risk and low-risk groups. This finding highlights the intricate and complex nature of tumor biology, suggesting that TMB may serve as an independent prognostic factor, especially within certain risk subgroups, thereby increasing variations in survival outcomes when used in conjunction with risk stratification methods. Notably, high TMB correlated with improved survival (p = 0.048), and combined TMB-risk stratification identified subgroups with distinct outcomes, suggesting TMB may serve as an independent prognostic factor when integrated with risk scores (42, 43).

Drug sensitivity predictions revealed the responsiveness of high-risk patients to targeted therapies (Afuresertib, Venetoclax, Navitoclax), which correlates with the activation of PI3K-AKT pathways and BIRC5 overexpression in these subgroups. This highlights the potential of precision oncology approaches, where high-risk patients may derive clinical benefit from targeted agents, while low-risk patients with an immunologically “hot” tumor microenvironment could be prioritized for immune checkpoint inhibitors (ICIs). Additionally, the underexplored role of the STING pathway in reshaping the immune microenvironment provides a promising avenue for combinatorial therapies in low-risk subgroups.

In addition to TMB and immune features, our study also explored the therapeutic implications of risk stratification through drug sensitivity analysis. We found that the high-risk group exhibited significantly increased sensitivity to aurorasertib, doramapimod, navitoclax, ribociclib, and venetoclax. These compounds target mitotic checkpoints, cell cycle regulators, and apoptosis pathways—mechanistically aligning with our functional enrichment results that revealed upregulation of proliferation- and stress-related pathways in high-risk tumors. This suggests that these patients may benefit more from therapies targeting proliferative and anti-apoptotic processes, providing a rationale for integrating our model into personalized treatment planning. To further validate the cellular origin and biological relevance of key prognostic genes, single-cell transcriptomic analysis performed. EZH2, PCNA, and BIRC5 were predominantly expressed in epithelial clusters, supporting their tumor-intrinsic roles (44). Additionally, LGALS9–CD44 emerged as a key ligand–receptor axis mediating interactions between monocytes and epithelial cells, implicating Galectin-9 in immunosuppressive signaling through CD8^+^ T cell inhibition (45). These observations are consistent with recent studies highlighting the role of tumor–immune crosstalk in modulating therapeutic responses (46, 47), and further underscore the translational potential of integrating single-cell analysis with risk modeling to inform immunotherapy strategies.

Notwithstanding these insights, a significant limitation is the lack of GTEx normal tissue data to complement TCGA’s tumor-normal comparisons. GTEx, a comprehensive gene expression resource encompassing various healthy tissues, is extensively utilized to differentiate cancer-specific expression alterations from tissue-specific baselines, thereby enhancing the specificity of differential expression gene (DEG) analysis. For instance, biomarkers such as EZH2 (high-risk) and CD34 (protective) would benefit from validation against GTEx-derived normal cervical tissue expression to confirm their cancer-specific dysregulation. While our DEG analysis employed existing TCGA tumor-normal pairs, it will be crucial for future studies to integrate GTEx’s larger and more diverse normal cohort in order to refine biomarker discovery and strengthen biological interpretability. This limitation underscores the necessity for multi-cohort validation that incorporates diverse normal tissue references to improve the generalizability of our findings. In addition to these considerations, another notable limitation arises from reliance on a single GEO dataset (GSE30759) for validation purposes, which may impact generalizability across varied populations or technical platforms. Although GSE30759 was chosen due to its substantial sample size and technical consistency with TCGA data, future investigations should aim to validate the model within multiple independent cohorts and among diverse clinical populations—including those representing different ethnic backgrounds or sequencing methodologies. Furthermore, the reliance of this study on computer simulation analysis, combined with the relatively limited sample size, imposes considerable limitations. And there may be batch effects between data sets, which may introduce variability and may also affect the reproducibility of our findings. In addition, the analysis flow of the study followed a conventional linear format and lacked novel algorithmic innovations or modular designs. While we currently lack the resources to implement advanced computational frameworks or perform wet laboratory validation, we are clearly committed to filling these gaps in future studies. Key steps will include: 1) validation with a larger multicenter dataset to mitigate batch effects and improve statistical power; 2) develop modular, open source libraries with adaptive machine learning algorithms to model gene-gene interactions; 3) Immunohistochemical staining of EZH2 and CXCL12 was performed in clinical tissue microarray to verify protein expression; 4) qPCR in paired normal tumor samples to validate the transcriptomics results; 5) perform functional experiments such as CRISPR-Cas9 knockout/overexpression experiments in cervical cancer cell lines to determine the role of biomarkers in tumor progression. By integrating computational prediction and mechanistic validation, these efforts will improve the translational relevance of our results and pave the way for personalized treatment strategies for cervical cancer.

## 5 Conclusion

In conclusion, this study provides valuable insights into cervical cancer, revealing several findings with significant clinical implications. The prognostic risk model developed from genes such as CHMP4C, ATP13A2, and ALDOA serves as a promising tool for predicting patient outcomes. Furthermore, the observed differences in immune cell infiltration between high- and low-risk groups indicate the potential for immune-based therapies. The TMB emphasizes the importance of genetic mutations as key biomarkers for prognosis and response to therapy. Although these results are encouraging, it is essential to validate them in diverse populations. Future research should aim to deepen our understanding of changes in the immune microenvironment and investigate targeted therapies to improve patient survival. Additionally, integrating multi-omics data will enhance risk models and facilitate the development of personalized treatments for cervical cancer.

## Data Availability

The original contributions presented in the study are included in the article/supplementary material. Further inquiries can be directed to the corresponding author.
